# Long-term multiple metabolic abnormalities among healthy and high-risk people following nonsevere COVID-19

**DOI:** 10.1038/s41598-023-41523-5

**Published:** 2023-08-31

**Authors:** Chaiwat Washirasaksiri, Naruemit Sayabovorn, Pinyapat Ariyakunaphan, Chayanis Kositamongkol, Thanet Chaisathaphol, Tullaya Sitasuwan, Rungsima Tinmanee, Chonticha Auesomwang, Pongpol Nimitpunya, Diana Woradetsittichai, Methee Chayakulkeeree, Pakpoom Phoompoung, Korapat Mayurasakorn, Nitat Sookrung, Anchalee Tungtrongchitr, Rungsima Wanitphakdeedecha, Saipin Muangman, Sansnee Senawong, Watip Tangjittipokin, Gornmigar Sanpawitayakul, Cherdchai Nopmaneejumruslers, Visit Vamvanij, Pochamana Phisalprapa, Weerachai Srivanichakorn

**Affiliations:** 1 Division of Ambulatory Medicine, Department of Medicine, Faculty of Medicine Siriraj Hospital, Mahidol University, 2 Wang Lang Road, Bangkok Noi, Bangkok, 10700 Thailand; 2 Division of Infectious Diseases and Tropical Medicine, Department of Medicine, Faculty of Medicine Siriraj Hospital, Mahidol University, Bangkok, Thailand; 3 Siriraj Population Health and Nutrition Research Group, Department of Research Group and Research Network, Faculty of Medicine Siriraj Hospital, Mahidol University, Bangkok, Thailand; 4 Center of Research Excellence On Therapeutic Proteins and Antibody Engineering, Department of Parasitology, Faculty of Medicine Siriraj Hospital, Mahidol University, Bangkok, Thailand; 5 Department of Parasitology, Faculty of Medicine Siriraj Hospital, Mahidol University, Bangkok, Thailand; 6 Department of Dermatology, Faculty of Medicine Siriraj Hospital, Mahidol University, Bangkok, Thailand; 7 Department of Anesthesiology, Faculty of Medicine Siriraj Hospital, Mahidol University, Bangkok, Thailand; 8 Department of Immunology, Faculty of Medicine Siriraj Hospital, Mahidol University, Bangkok, Thailand; 9 Division of Ambulatory Paediatrics, Department of Paediatrics, Faculty of Medicine Siriraj Hospital, Mahidol University, Bangkok, Thailand; 10 Department of Nursing, Faculty of Medicine Siriraj Hospital, Mahidol University, Bangkok, Thailand; 11 Department of Orthopaedic Surgery, Faculty of Medicine Siriraj Hospital, Mahidol University, Bangkok, Thailand

**Keywords:** Diabetes, Dyslipidaemias, Metabolic syndrome, Obesity, Pre-diabetes

## Abstract

Few studies have identified the metabolic consequences of the post-acute phase of nonsevere COVID-19. This prospective study examined metabolic outcomes and associated factors in nonsevere, RT-PCR-confirmed COVID-19. The participants’ metabolic parameters, the prevalence of long-term multiple metabolic abnormalities (≥ 2 components), and factors influencing the prevalence were assessed at 1, 3, and 6 months post-onset. Six hundred individuals (mean age 45.5 ± 14.5 years, 61.7% female, 38% high-risk individuals) with nonsevere COVID-19 attended at least one follow-up visit. The prevalence of worsening metabolic abnormalities was 26.0% for BMI, 43.2% for glucose, 40.5% for LDL-c, 19.1% for liver, and 14.8% for C-reactive protein. Except for lipids, metabolic-component abnormalities were more prevalent in high-risk hosts than in healthy individuals. The prevalence of multiple metabolic abnormalities at the 6-month follow-up was 41.3% and significantly higher in high-risk than healthy hosts (49.2% vs 36.5%; *P* = 0.007). Factors independently associated with a lower risk of these abnormalities were being female, having dyslipidemia, and receiving at least 3 doses of the COVID-19 vaccine. These findings suggest that multiple metabolic abnormalities are the long-term consequences of COVID-19. For both high-risk and healthy individuals with nonsevere COVID-19, healthcare providers should monitor metabolic profiles, encourage healthy behaviors, and ensure complete vaccination.

## Introduction

The coronavirus disease 2019 (COVID-19) pandemic, caused by severe acute respiratory syndrome coronavirus 2 (SARS-CoV-2) infection, is a global health threat. As at June 28, 2023, the pandemic has resulted in approximately 767 million global cases and 6.9 million deaths^1^. SARS-CoV-2 continues to spread worldwide and is a major public and international concern. SARS-CoV-2 can affect health in a wide range of presentations^2^. The severity of symptoms depends on several factors related to the infected individuals, such as age, comorbidities, and vaccination status^3–5^. Although the respiratory tract is the leading infected site of SARS-CoV-2, the virus can involve many other systems in the human body^6^. The effects of SARS-CoV-2 infections on the body are related to the binding of the angiotensin-converting enzyme 2 (ACE2) receptor in the cell membranes of many organs, such as the lungs, heart, gastrointestinal tract, liver, kidney, and pancreas^7,8^. The coronavirus uses a spike protein on its surface to bind with the ACE2 receptor, enabling the virus to enter the cell by endocytosis^8^.

Previous studies on severe cases of COVID-19 have demonstrated that SARS-CoV-2 infection has both short-term and long-term health consequences. During the infectious period, individuals may experience respiratory tract abnormalities and worsened overall health status due to multiorgan dysfunction triggered by a cytokine response known as a cytokine storm^9,10^. In the long term, persistent abnormal symptoms, pathological chest imaging findings, and deterioration of metabolic parameters have been reported^11,12^. However, there is a lack of information regarding the health consequences, particularly the metabolic aspects, in nonsevere cases following the onset of COVID-19^13,14^.

Metabolic processes play a crucial role in the pathogenesis, pathophysiology, and host response to SARS-CoV-2 infection, as well as the effectiveness of vaccines against SARS-CoV-2 infection^15,16^. Therefore, metabolic profiles, inflammatory markers, and liver function tests have been investigated^17–19^. Given the presence of ACE2 receptors in the pancreas, visceral adipose tissue, and other organs involved in metabolic regulation, metabolic disturbances could be potential sequelae in individuals who have recovered from or survived a SARS-CoV-2 infection^20–22^. During the acute phase of SARS-CoV-2 infections, many inflammatory markers of serious illness, such as the erythrocyte sedimentation rate, C-reactive protein (CRP), and procalcitonin, are elevated^23^. Similarly, abnormal liver function test results appear during SARS-CoV-2 infections due to several causes, such as direct virus invasion, medication effects, and underlying liver disease^24^. After recovery from COVID-19, inflammatory markers and liver enzymes either return to normal levels or persist at abnormal levels.

To date, investigations of the residual symptoms, inflammation, and some metabolic aspects of post-COVID-19 patients have focused on severe cases. For nonsevere cases of COVID-19, however, there is little information on the long-term consequences and the impact of host status on these relationships. The present analysis explored the following: (1) the prevalence of long-term multiple metabolic abnormalities and (2) the extent to which individual characteristics, host status, and immunization status are related to the metabolic consequences 3 and 6 months after recovery from nonsevere COVID-19.

## Results

In all, 5059 people with nonsevere COVID-19 were registered in the out-of-hospital isolation system of the Faculty of Medicine Siriraj Hospital, Thailand. Of those, 600 people aged between 18 and 85 with PCR-confirmed COVID-19 were eligible for our analyses. Most of the 600 participants attended the 3-month follow-up (587; 97.8%) and the 6-month follow-up (466; 77.7%). Overall, they had a mean age of 45.5 ± 14.5 years, BMI of 25.4 ± 5.0 kg/m^2^, and N-gene cycle threshold of 21.4 ± 5.8. More than half were women (370/600; 61.7%). Five hundred forty participants (90.0%) were symptomatic, and 577 (96.2%) were prescribed favipiravir during isolation. The proportion of fully vaccinated participants was low: 120 (20%) had received 2 vaccine doses before disease onset, while only 41 (6.8%) had received 3 vaccine doses. Almost all participants (568/600; 95%) fully recovered at home or in a hospital without needing hospital admission. However, 20 participants (3.2%) were reinfected by COVID-19 during the 6-month follow-up. Two hundred twenty-nine (38%) participants were classified as high-risk hosts. The demographic data and clinical outcomes of the healthy and high-risk hosts are summarized in Table 1. Overall, the high-risk host group had a significantly higher mean age, BMI, and proportion of COVID-19 reinfections than the healthy-host group. Conversely, the high-risk host group had a significantly lower proportion of symptomatic disease, fully immunized individuals, and fully recovered patients than the healthy hosts.

**Table 1:** Clinical baseline characteristics, comorbidities, and outcomes of healthy and high-risk hosts with nonsevere COVID-19.

Variables	Healthy hosts (n = 371)	High-risk hosts (n = 229)	*P*
Female, n (%)	239 (64.4%)	131 (57.2%)	0.07
Age, mean (± SD)	40.84 ± 11.95	52.98 ± 15.13	< 0.001
BW (kg)	62.10 ± 11.30	74.33 ± 18.46	< 0.001
BMI (kg/m^2^)	23.60 ± 3.41	28.27 ± 5.86	< 0.001
BMI < 25, n (%)	243 (65.5%)	72 (31.4%)	< 0.001
BMI 25–29.9	128 (34.5%)	61 (26.6%)	
BMI ≥ 30	-	96 (41.9%)	
N-gene CT threshold	21.41 ± 5.65	21.41 ± 5.92	0.9
High-risk for COVID-19, n (%)			
- Age ≥ 60	–	105 (45.9%)	< 0.001
CKD	–	14 (6.1%)	< 0.001
CAD	–	19 (8.3%)	< 0.001
CVD	–	3 (1.3%)	0.02
Chronic lung disease	–	11 (4.8%)	< 0.001
Previous DM	–	66 (28.8%)	< 0.001
Obese (BMI ≥ 30 kg/m^2^)	–	96 (41.9%)	< 0.001
Symptomatic*, n (%)	346 (93.3%)	194 (84.7%)	0.001
Immunization status**, n (%)			
Not immunized	139 (37.5%)	81 (35.4%)	< 0.001
Partially immunized	112 (30.2%)	107 (46.7%)	
Fully immunized (2 doses)	86 (23.2%)	34 (14.8%)	
Fully immunized (3 doses)	34 (9.2%)	7 (3.1%)	
Discharge status, n (%)			
Recovered	356 (96.0%)	212 (92.6%)	0.03
Referred to hospital	9 (2.4%)	15 (6.6%)	
Transferred to other hospital	6 (1.6%)	2 (0.9%)	
Reinfection with SARS-CoV-2, n (%)	11 (3.0%)	9 (3.9%)	0.5

*Asymptomatic people were patients without any common COVID-19 symptoms. **(1) Not immunized: people without any COVID-19 vaccine administration and those vaccinated within 14 days before the onset of COVID-19; (2) partially immunized: people who received only 1 vaccine dose more than 14 days before the onset of COVID-19; (3) fully immunized: people who received at least 2 vaccine doses more than 14 days before the onset of COVID-19. CAD, coronary artery disease; CKD, chronic kidney disease; CVD, cardiovascular disease; CT, cycle threshold; DM, diabetes mellitus; BMI, body mass index; BW, body weight; SD, standard deviation.

### Body weight and body mass index

**Figure 1:**
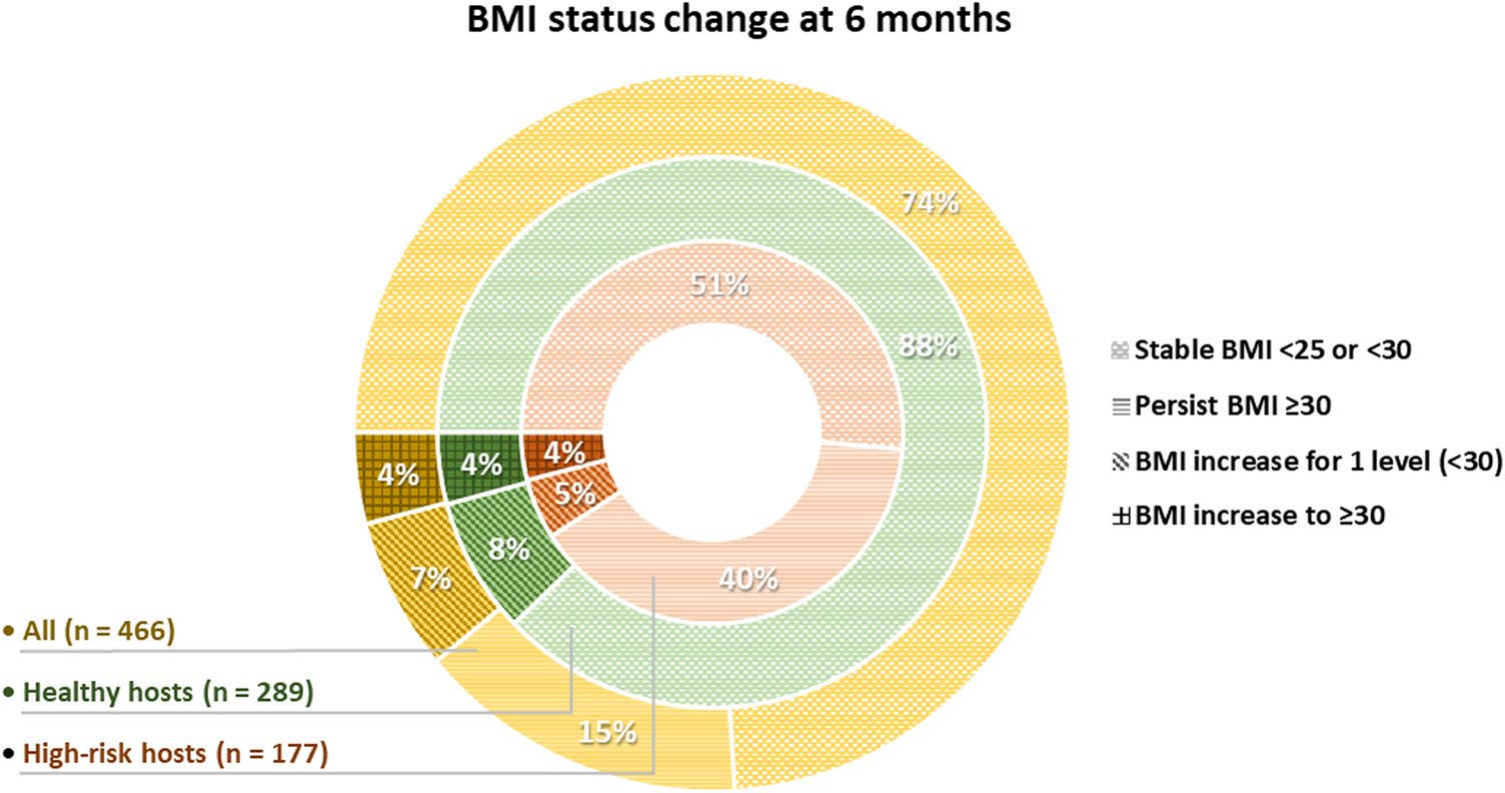
Changes in BMI statuses of the overall cohort, healthy hosts, and high-risk hosts from baseline to 6 months after COVID-19 infection.

Mean body weight increased significantly: by 0.88 ± 0.17 kg (*P* < 0.001) at 3 months and another 1.18 ± 0.19 kg (*P* < 0.001) at 6 months (Table 2). Consequently, the BMI showed corresponding significant increases: 3 months, 0.34 ± 0.06 kg/m^2^ (*P* < 0.001), and 6 months, 0.44 ± 0.07 kg/m^2^ (*P* < 0.001). The degrees of weight and BMI changes were similar for the healthy and high-risk hosts. Eleven percent of participants saw a BMI increase of at least 1 level from baseline (i.e., from a BMI of < 25 to a BMI of 25–< 30 kg/m^2^, and from a BMI of 25–< 30 kg/m^2^ to a BMI ≥ 30 kg/m^2^). These proportions were relatively higher in healthy hosts (12.2%) than in high-risk hosts (9.1%). In people with a baseline BMI ≥ 30, the proportion showing an increase in BMI was 10.5% (49/470) for overall participants but 27.6% (49/179) for high-risk participants (Fig. 1; Table 3). However, the proportion of people with a stable BMI at 6 months (i.e., remaining in the same BMI category or having a baseline BMI ≥ 30 with a subsequent decrease) was 78.5% (healthy hosts, 87.9%; high-risk hosts, 63.3%). Overall, the prevalence of long-term BMI abnormalities was 21.5%. It was significantly higher for high-risk hosts (36.7%) than for healthy hosts (12.1%; *P* < 0.001; Table 3).

**Table 2:** Metabolic changes 3 and 6 months after the onset of nonsevere COVID-19 among healthy and high-risk hosts.

Parameters	Baseline (N = 600)	3 months (N = 587)	6 months (N = 466)	Δ Change ± SE (*p*)	
BW (mean ± SE)	Baseline	3 months	6 months	ΔBW B-3 m ± SE (*P*)	ΔBW B-6 m ± SE (*P*)
All (n = 457)	68.6 ± 0.69	69.5 ± 0.72	69.8 ± 0.73	− 0.88 ± 0.17 (< 0.001)	− 1.18 ± 0.19 (< 0.001)
Healthy Hosts (n = 284)	62.1 ± 0.85	63.2 ± 0.89	63.3 ± 0.90	− 1.06 ± 0.20 (< 0.001)	− 1.21 ± 0.23 (< 0.001)
High-risk hosts (n = 173)	75.1 ± 1.09	75.8 ± 1.14	76.3 ± 1.16	− 0.70 ± 0.26 (0.023)	− 1.16 ± 0.29 (< 0.001)
BMI (mean ± SE)	Baseline	3 months	6 months	ΔBMI B-3 m ± SE (*P*)	ΔBMI B-6 m ± SE (*P*)
All (n = 457)	25.5 ± 0.24	25.9 ± 0.24	26.0 ± 0.25	− 0.34 ± 0.06 (< 0.001)	− 0.44 ± 0.07 (< 0.001)
Healthy hosts (n = 284)	23.7 ± 0.27	24.1 ± 0.28	24.2 ± 0.28	− 0.40 ± 0.08 (< 0.001)	− 0.45 ± 0.09 (< 0.001)
High-risk hosts (n = 173)	28.5 ± 0.35	28.8 ± 0.36	29.0 ± 0.36	− 0.25 ± 0.10 (0.012)	− 0.43 ± 0.11 (< 0.001)
HbA1c (mean ± SE)		3 months	6 months	ΔHbA1c 3 m-6 m ± SE (*P*)	
All (n = 464)		5.9 ± 0.05	6.0 ± 0.05	− 0.09 ± 0.02 (< 0.001)	
Healthy hosts (n = 285)		5.7 ± 0.06	5.8 ± 0.07	− 0.07 ± 0.02 (0.001)	
High-risk hosts (n = 179)		6.0 ± 0.08	6.1 ± 0.08	− 0.10 ± 0.03 (< 0.001)	
LDL (mean ± SE)		3 months	6 months	ΔLDL3m-6 m ± SE (*P*)	
All (n = 465)		126.1 ± 1.74	122.2 ± 1.68	3.87 ± 0.99 (< 0.001)	
Healthy hosts (n = 287)		129.7 ± 2.15	126.8 ± 2.08	2.92 ± 1.22 (0.01)	
High-risk hosts (n = 178)		122.4 ± 2.73	117.6 ± 2.64	4.82 ± 1.55 (0.002)	
AST (mean ± SE)		3 months	6 months	ΔAST3m-6 m ± SE (*P*)	
All (n = 466)		22.8 ± 0.52	24.0 ± 2.77	− 1.17 ± 1.76 (0.5)	
Healthy hosts (n = 288)		22.1 ± 0.66	21.5 ± 2.25	0.56 ± 2.24 (0.8)	
High-risk hosts (n = 178)		24.1 ± 0.84	28.1 ± 2.86	− 3.98 ± 2.85 (0.1)	
ALT (mean ± SE)		3 months	6 months	ΔALT3m-6 m ± SE (*P*)	
All (n = 466)		23.3 ± 0.70	23.6 ± 1.92	− 0.25 ± 1.88 (0.8)	
Healthy hosts (n = 288)		22.0 ± 0.89	20.4 ± 2.44	1.67 ± 2.40 (0.4)	
High-risk hosts (n = 178)		25.4 ± 1.13	28.7 ± 3.10	− 3.36 ± 3.04 (0.2)	
CRP (mean ± SE)		3 months	6 months	ΔCRP3m-6 m ± SE (*P*)	
All (n = 446)		3.5 ± 0.40	3.2 ± 0.25	0.32 ± 0.43 (0.4)	
Healthy hosts (n = 288)		2.5 ± 0.51	3.0 ± 0.32	− 0.44 ± 0.55 (0.4)	
High-risk hosts (n = 178)		5.1 ± 0.64	3.6 ± 0.41	1.56 ± 0.70 (0.02)	

*ALT* alanine aminotransferase, *AST* aspartate aminotransferase, *B-3m* baseline to 3 months, *3m–6m* 3 months to 6 months, *BW* body weight, *BMI* body mass index, *CRP* c-reactive protein, *LDL* low-density lipoprotein cholesterol, *SE* standard error.

**Table 3:** The prevalence of “overall multiple metabolic abnormalities” and of “each metabolic component” among healthy and high-risk hosts.

Multiple metabolic abnormality	All (n = 470)	Healthy hosts (n = 291)	High-risk hosts (n = 179)	*P*
**Abnormality in BMI component, n (%)**	100 (21.5%)	35 (12.1%)	65 (36.7%)	< 0.001
-Worsening BMI to 25–< 30 kg/m^2^	32 (6.9%)	23 (8.0%)	9 (5.1%)	< 0.001
-Worsening BMI to ≥ 30 kg/m^2^	19 (4.1%)	12 (4.2%)	7 (4.0%)	
-High BMI ≥ 30 kg/m^2^ with increased BMI	49 (10.5%)	–	49 (27.6%)	
**Abnormality in glucose homeostasis, n (%)**	206 (43.1%)	119 (40.3%)	87 (47.8%)	0.1
-HbA1c 5.7–6.4	168 (35.2%)	101 (34.2%)	67 (36.8%)	0.05
-Newly diagnosed diabetes	35 (7.3%)	18 (6.1%)	17 (9.3%)	
- Known DM with uncontrolled HbA1c at 6 months (HbA1c ≥ 9%)	3 (0.6%)	–	3 (1.6%)	
**Abnormality in lipid component, n (%)**	190 (40.5%)	132 (45.5%)	58 (32.4%)	0.005
-Persistent hypercholesterolemia (d-LDL ≥ 130 mg/dL at 6 m)	32 (6.8%)	24 (8.3%)	8 (4.5%)	0.1
-Known dyslipidemia with uncontrolled LDL (d-LDL ≥ 130 mg/dL at 6 m)	158 (33.7%)	108 (37.2%)	50 (27.9%)	
**Abnormality in liver enzyme component, n (%)**	89 (19.1%)	45 (15.6%)	44 (24.7%)	0.01
-Worsening of AST, ALT, or both to > 30 U/L at 6 m	32 (6.9%)	14 (4.9%)	18 (10.1%)	0.03
-Persistent abnormality (≥ 30 U/L) of AST, ALT, or both at 6 m	57 (12.2%)	31 (10.8%)	26 (14.6%)	
**Abnormality in CRP component, n (%)**	69 (14.8%)	36 (12.5%)	33 (18.5%)	0.07
-Persistent CRP abnormality ≥ 5 mg/L	42 (9.0%)	22 (7.6%)	20 (11.2%)	0.2
-Worsening to CRP ≥ 5 mg/L	27 (5.8%)	14 (4.9%)	13 (7.3%)	
**Multiple metabolic abnormality, n (%**)	192 (41.3%)	105 (36.5%)	87 (49.2%)	0.007

*ALT* alanine aminotransferase, *AST* aspartate aminotransferase, *BMI* body mass index, *CRP* c-reactive protein, *d-LDL* direct low-density lipoprotein cholesterol, *DM* diabetes mellitus, *HbA1c* hemoglobin A1c, *LDL* low-density lipoprotein cholesterol, *m* month.

### Glycemic change, HbA1c prediabetes, and newly diagnosed diabetes

**Figure 2:**
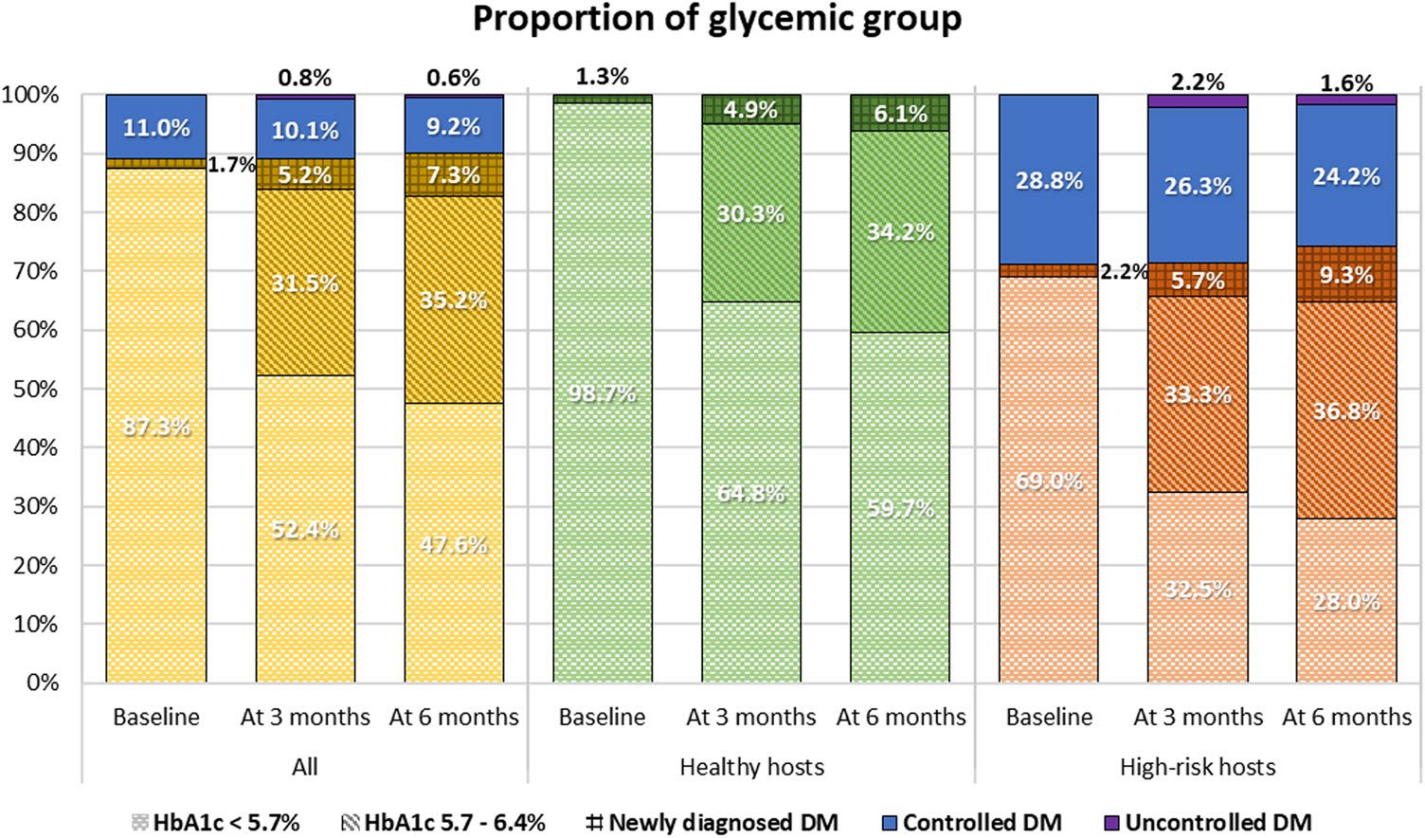
Changes in glycemic statuses of the overall cohort, healthy hosts, and high-risk hosts from baseline to 3 and 6 months after COVID-19 infection.

At baseline, the prevalence of people without diabetes was 87.3% (n = 524) and with diabetes was 12.7% (n = 76). Of the 76 diabetics, 66 (11%) had known diabetes before their COVID infection. The remaining 10 patients (1.7%) were newly diagnosed with diabetes upon admission to the out-of-hospital isolation system. The mean change in HbA1c of overall participants increased significantly from 5.9 ± 0.05% at the 3-month visit to 6.0 ± 0.05% at the 6-month visit (*P* < 0.001). An increasing trend in mean HbA1c was found in healthy and high-risk hosts (healthy hosts, 0.07 ± 0.02% [*P* < 0.001]; high-risk hosts, 0.10 ± 0.03% [*P* < 0.001]; Table 2). Moreover, the cumulative prevalence of prediabetes (HbA1c 5.7–6.4%) rose from 31.5% at the 3-month visit to 35.2% at the 6-month visit. Similarly, the cumulative prevalence increased from 16.1 to 17.1% among all people with diabetes and from 5.2 to 7.3% among newly diagnosed diabetics (Fig. 2). Furthermore, among high-risk hosts, the proportion with newly diagnosed diabetes 6 months after their COVID-19 infection (9.3%) was higher than that for healthy hosts (6.1%).

Overall, the prevalence of long-term abnormal glucose homeostasis increased over time and reached 43.1% at 6 months. However, there was no significant difference in the proportion of individuals in the healthy and high-risk host groups with long-term abnormal glucose homeostasis (40.3% and 47.8%, respectively; *P* = 0.1; Table 3).

### Lipid level and hypercholesterolemia status

**Figure 3:**
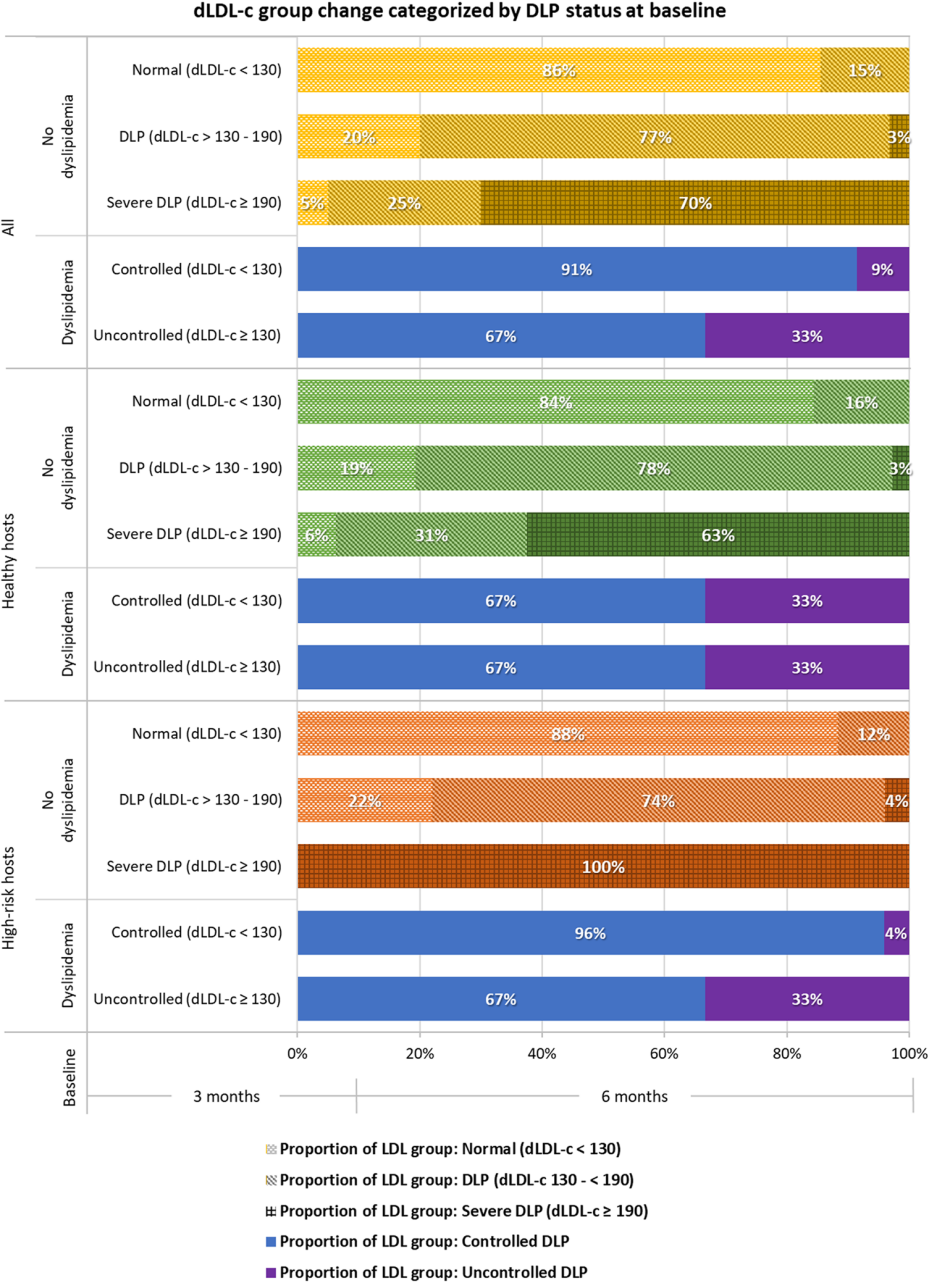
Changes in lipid statuses of known and newly detected cases of dyslipidemia in the overall cohort, healthy hosts, and high-risk hosts from 3 to 6 months after COVID-19 infection.

The prevalence of known hypercholesterolemia was 16.8% (n = 102). The cumulative prevalence of hypercholesterolemia (both known and newly detected dyslipidemia [dLDL-c ≥ 130 mg/dL]) was 56.5% (n = 337) at 3 months and 67.3% (n = 369) at 6 months. However, both groups saw the mean dLDL-c fall significantly between 3 and 6 months (healthy hosts, by 2.92 ± 1.22 mg/dL [*P* = 0.01]; high-risk hosts, by 4.82 ± 1.55 [*P* = 0.002]; Table 2). In participants with no known history of dyslipidemia, 15% experienced an increase in their dLDL-c level from a normal value at 3 months to ≥ 130 mg/dL at 6 months. The proportion of people who maintained a high dLDL-c level (130–< 190 mg/dL) at their 6-month visit was 77%. The proportions of people at the 6-month visits whose dLDL-c levels had risen to ≥ 190 mg/dL or remained high (≥ 190 mg/dL) were 3% and 70%, respectively. For people with dyslipidemia prior to their COVID-19 infection, the proportions with uncontrolled dLDL-c or deterioration in uncontrolled dLDL-c at 6 months were 33% and 9%, respectively (Fig. 3). Overall, 40.5% of the study cohort had abnormal lipid profiles at 6 months. Moreover, the proportion of healthy hosts with lipid abnormalities was significantly higher than that of high-risk hosts (45.5% vs 32.4%; *P* = 0.005; Table 3).

### Liver abnormality

**Figure 4:**
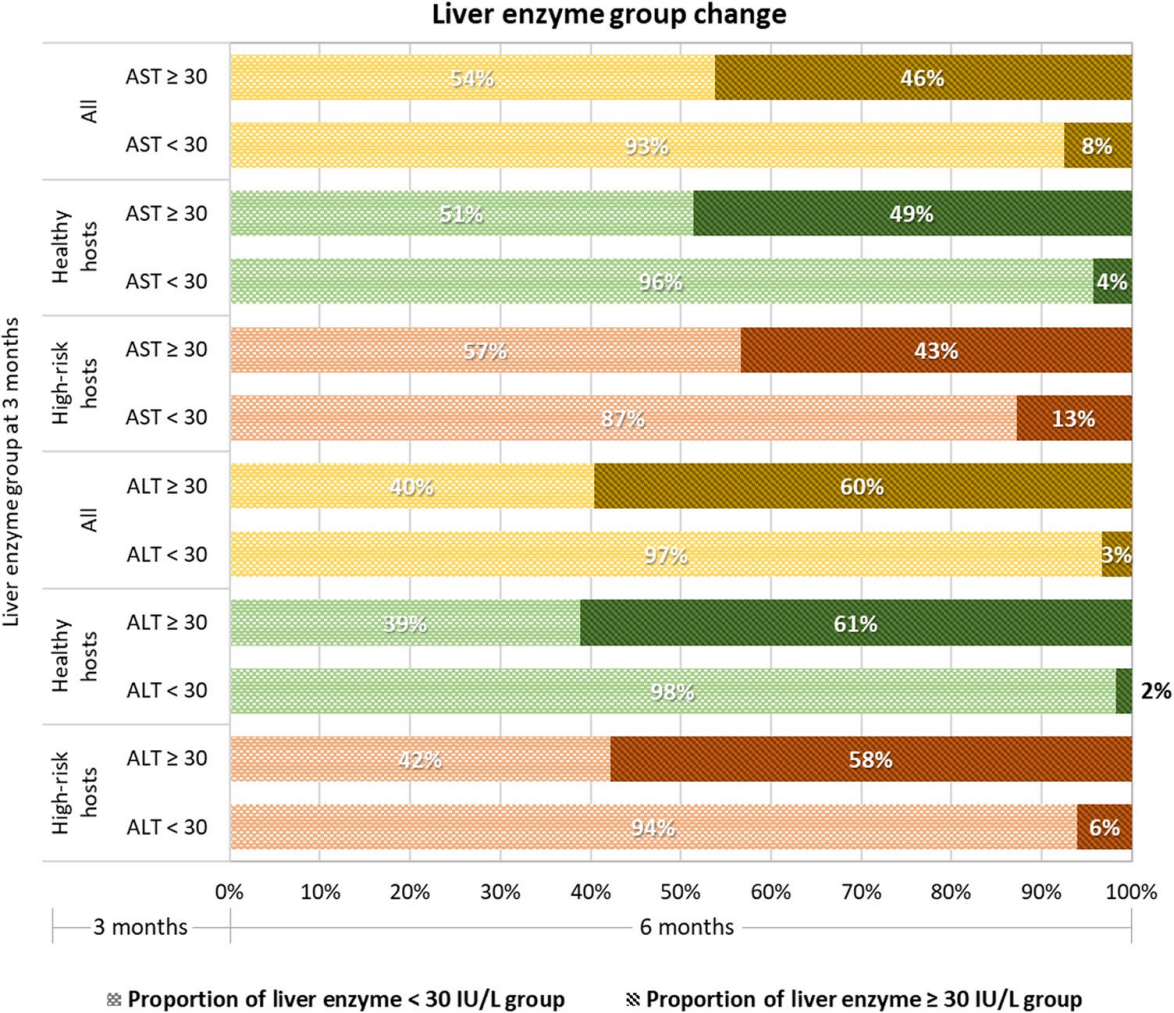
Changes in AST and ALT statuses of the overall cohort, healthy hosts, and high-risk hosts from 3 to 6 months after COVID-19 infection.

There were no differences in the mean levels and mean changes in AST and ALT at 3 and 6 months. However, the prevalence of abnormal AST (≥ 30 U/L) or ALT (≥ 30 U/L) was 25% at the 3-month visit (Tables 2, 3). At the 6-month visit, 69.7% of people maintained normal liver function, 11.2% showed improved liver function, and 19.1% maintained abnormal liver function (worsened for 6.9% but stable for 12.2%). The proportions with abnormal AST and ALT or deterioration in abnormal AST and ALT at 6 months are illustrated in Fig. 4. Overall, the prevalence of long-term abnormal liver function was 19.1%. The high-risk host group had a significantly higher proportion of people with abnormal liver function (24.7%) than the healthy-host group (15.6%; *P* = 0.01).

### CRP level abnormality

**Figure 5:**
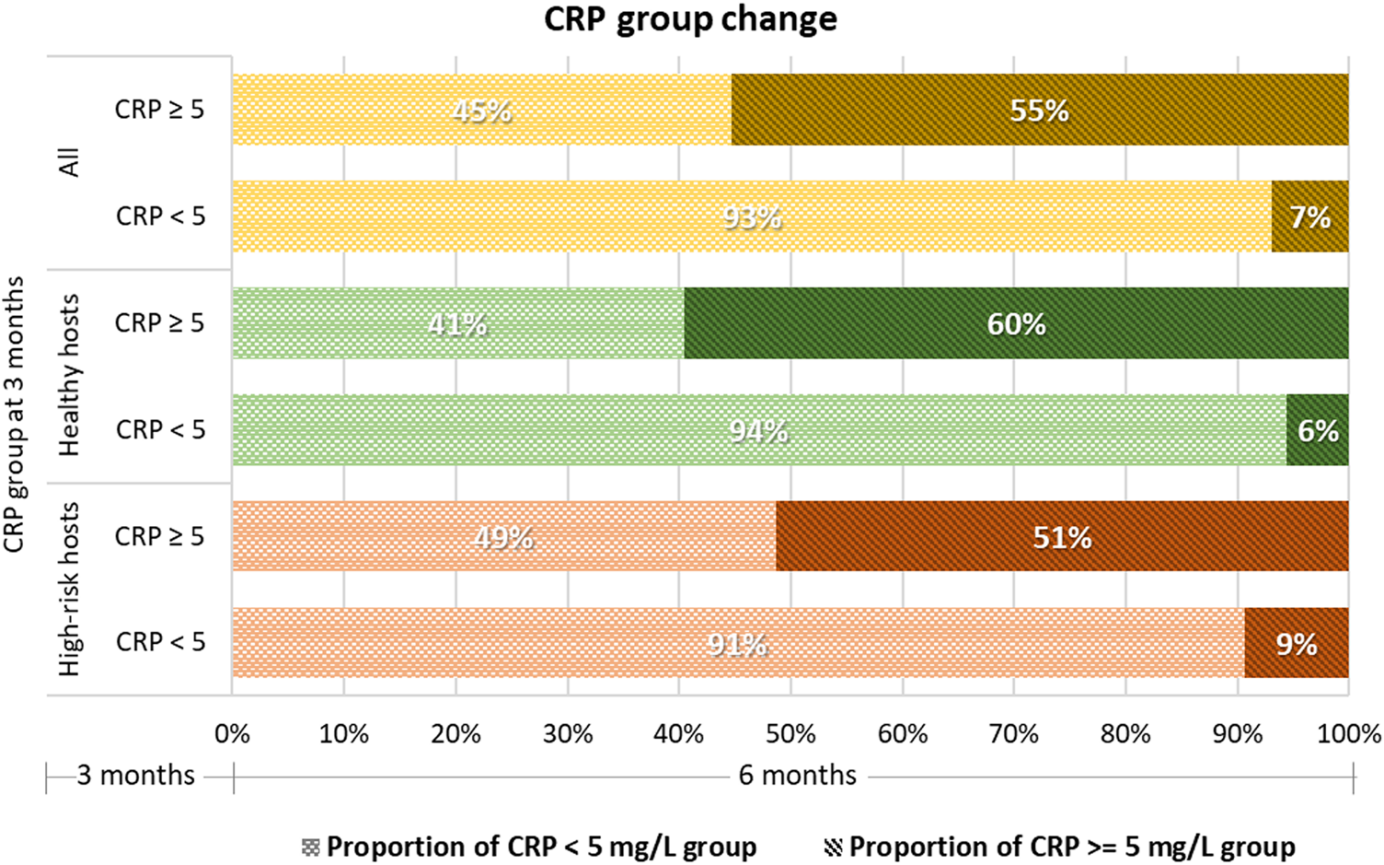
Changes in CRP statuses of the overall cohort, healthy hosts, and high-risk hosts from 3 to 6 months after COVID-19 infection.

The overall participant group and the healthy hosts showed no differences in the mean changes to their respective CRP levels at the 3- and 6-month visits. However, the mean CRP level of the high-risk hosts fell significantly between the 3- and 6-month visits, falling by 1.56 ± 0.70 mg/L (*P* = 0.02). The mean CRP level for the 3-month visit was 5.1 ± 0.6 mg/L, while the mean for the 6-month visit was 3.6 ± 0.4 mg/L (Table 2). The proportion of people with abnormal CRP levels (≥ 5 mg/L) at the 3-month visit was 16.3%, with 14.8% continuing to have abnormal levels at 6 months. The proportion with abnormal CRP or deterioration in CRP at 6 months is depicted in Fig. 5. Overall, the prevalence of long-term CRP abnormalities was 14.8%. There was no significant difference between the high-risk and healthy hosts (18.5% vs 12.5%; *P* = 0.07).

### Factors predicting long-term multiple metabolic abnormalities

The prevalence of long-term multiple metabolic abnormalities at the 6-month visit was 41.3%. The prevalence was significantly higher for high-risk hosts (49.2%) than for healthy hosts (36.5%; *P* = 0.007). Compared with individuals who did not have multiple metabolic abnormalities (Supplementary Table 1), those with multiple abnormalities had the following:

- a significantly higher proportion of men (45.3% vs 33.0%)
- a significantly higher proportion of high-risk hosts (45.3% vs 33.0%)
- a significantly higher mean BMI (27.5 ± 5.0 kg/m^2^ vs 24.2 ± 4.7 kg/m^2^)
- a significantly lower proportion of individuals with known dyslipidemia (10.9% vs 21.2%)

As also detailed in Supplementary Table 1, there were no significant differences in the following parameters of people with and without multiple metabolic abnormalities:

- their baseline mean ages and N-gene cycle thresholds
- their baseline median utility and VAS scores
- the proportions with hypertension at baseline
- the proportions with symptoms at the onset of infection
- the proportions who fully recovered at home or in a hospital
- their baseline immunization statuses
- their vaccine booster statuses after COVID-19 onset

Independent prognostic factors of multiple metabolic abnormalities were explored. Our multivariable logistic regression models examined gender, duration of antiviral medication use (favipiravir), hypertension, dyslipidemia, high-risk status for COVID-19 complications, and immunization status. Overall, the factors found to be independently associated with a lower risk of progressing to multiple metabolic abnormalities were the following:

- being female rather than male (adjusted odds ratio [aOR] 0.64; 95% confidence interval [CI] 0.43–0.96); *P* < 0.03)
- having dyslipidemia rather than being without dyslipidemia (aOR 0.27; 95% CI 0.14–0.55); *P* < 0.001)
- receiving at least 3 doses of any COVID-19 vaccine rather than being without previous vaccination before the onset of COVID-19 (aOR 0.39, 95% CI 0.15–0.98, *P* = 0.04)

Participants with any of the features that characterized a high-risk host had a greater risk of progressing to multiple metabolic abnormalities than participants with the features of a healthy host (aOR 2.18; 95% CI 1.38–3.46; *P* = 0.001; Table 4).

**Table 4:** Crude and adjusted odds ratios of multiple metabolic abnormalities within 6 months for each relevant factor among people with nonsevere COVID-19.

Variables	Univariable analysis		Multivariable analysis	
	OR (95% CI)	*P*	aOR (95% CI)	*P*
Female	0.59 (0.41–0.89)	0.007	0.64 (0.43–0.96)	0.03
Duration of favipiravir	1.10 (0.97–1.24)	0.1	1.09 (0.95–1.24)	0.2
Comorbidities				
HT	0.82 (0.53–1.25)	0.3	0.94 (0.54–1.71)	0.9
DLP	0.46 (0.27–0.78)	0.004	0.27 (0.14–0.55)	< 0.001
High-risk COVID-19 hosts	1.69 (1.15–2.46)	0.007	2.18 (1.38–3.46)	0.001
Immunization status				
Not immunized	1		1	
Partially immunized	0.85 (0.55–1.32)	0.4	0.91 (0.57–1.44)	0.6
Fully immunized (2 doses)	0.71 (0.43–1.18)	0.1	0.80 (0.47–1.37)	0.4
Fully immunized (3 doses)	0.31 (0.13–0.76)	0.01	0.39 (0.15–0.98)	0.04

*aOR* adjusted odds ratio, *CI* confidence interval, *DLP* dyslipidemia, *HT* hypertension, *OR* odds ratio. In addition to those shown, the predictor variables in each multivariable model included sex, duration of favipiravir, HT status, DLP status, high-risk COVID-19 host status, and immunization status. The multiple logistic regression equation was as follows: having long-term multiple metabolic abnormalities =  − 0.47 − (0.45 × female) + (0.08 × duration of favipiravir [days]) + 0.78 (being high-risk host) − (0.04 × HTN) − (1.30 × DLP) − (0.10 × partially immunized) − (0.22 × fully immunized [2 doses]) − (0.95 × fully immunized [3 doses]).

## Discussion

We explored the metabolic consequences of nonsevere COVID-19 that are apparent 3 and 6 months after disease onset and the impact of hosts’ clinical characteristics on these consequences. The study recruited 600 participants: 229 with high-risk features for COVID-19 complications (high-risk hosts) and 371 without high-risk features (healthy hosts). Smaller proportions of the high-risk hosts had symptomatic presentations, complete immunization, and full recovery at home than the healthy hosts. We found that 6 months after COVID-19 onset, the participants demonstrated significantly increased mean values for body weight, BMI, and HbA1c; a decreased mean dLDL-c level; and constant mean AST, ALT, and CRP levels. The healthy and high-risk host subgroups had similar mean changes to the overall cohort. Compared with healthy hosts, the high-risk hosts had significantly higher prevalences of the BMI and liver components of long-term multiple metabolic abnormalities but a lower prevalence for the lipid component. A lower risk of multiple metabolic abnormalities was associated with being female, having dyslipidemia, being fully immunized with at least 3 doses of any COVID-19 vaccine, and being a healthy host.

In parallel with our metabolic findings, COVID-19 recovery has various consequences, particularly in severe cases^25,26^. Studies have reported weight loss in hospitalized and non-hospitalized patients during COVID-19 illness and recovery^27,28^. However, our study revealed significant weight gain in most nonsevere COVID-19 cases, especially among healthy individuals. Post-COVID-19 recovery has been linked to new-onset type 2 diabetes or persistent hyperglycemia in nondiabetic individuals^29–32^. We found a 6-month prevalence of newly diagnosed diabetes of 7.3%, lower than the rate of 14% in predominantly hospitalized cases reported by a meta-analysis^33^. Prediabetes (HbA1c 5.7–6.4%) was observed in approximately one-third of our participants, twice the general population rate^34^. Furthermore, our study showed that 40.5% of patients had worsened serum lipid levels after 6 months. This finding aligns with a study in Italy, which observed significant increases in total cholesterol, high-density lipoprotein cholesterol, LDL cholesterol, and triglycerides in hospitalized patients 1 month after infection^35^. A metabolomic study in China showed that individuals with severe acute respiratory syndrome (SARS) exhibited elevated lipid metabolites and metabolic disturbances^26^. However, studies focusing on nonsevere COVID-19 cases for long-term outcomes are limited. A recent controlled study reported higher risks and burdens of dyslipidemia even 1 year after COVID-19 onset, compared to contemporary non-COVID controls^36^. These findings align with our observations, highlighting the impact of COVID-19 on lipid deterioration. Disparities in outcomes between healthy and high-risk individuals may be attributed to group-specific characteristics.

These metabolic findings suggest that individuals with nonsevere COVID-19 may experience minimal long-term adverse effects on their appetite and other medical conditions than those with severe disease. During isolation, people tend to engage in more sedentary behaviors and consume more high-energy-dense, sugary, and fatty foods and snacks^37–39^. Studies have shown that COVID-19-related lockdowns have led to weight gain among individuals^39,40^. These unhealthy lifestyles can impact the weight, glycemic control, lipid profile, and other metabolic factors of COVID-19-infected participants.

Furthermore, existing literature suggests a bidirectional relationship between COVID-19 and metabolic abnormalities^16,22,41,42^. SARS-CoV-2 can increase inflammatory cytokines in metabolism-related organs, particularly the pancreas and visceral adipose tissue. This affects beta-cell function, promotes toxicity to islet cells, induces beta-cell apoptosis, and triggers adipose tissue inflammation. These processes contribute to insulin resistance, hyperinsulinemia, elevated glycemic levels, nonalcoholic fatty liver disease, and alterations in hepatic lipoprotein metabolism and gut microbiome^22,42–46^

Our study highlights the importance of healthy and high-risk individuals with nonsevere COVID-19 being made aware of the risk of developing metabolic abnormalities after recovery. SARS-CoV-2 has the potential to cause metabolic dysregulation. Its presence within a community can also lead to unhealthy behaviors that contribute to abnormal metabolism and unfavorable weight gain, glycemic control, and lipid profile outcomes for both healthy and high-risk individuals.

Additionally, abnormal liver function is a significant concern for physicians during COVID-19, with the liver being the second most affected organ after the lungs^17,47,48^. Multiple factors contribute to liver abnormalities in COVID-19 patients, including direct viral invasion^49^, the individuals’ clinical characteristics and underlying liver disease^50,51^, disease severity^18,52^, subsequent development of nonalcoholic fatty liver disease^42^, and medications administered during and after hospitalization^53^. Previous research has shown a higher prevalence of abnormal liver function tests in severe COVID-19 cases than in nonsevere cases^54,55^. However, limited studies have explored the long-term liver function outcomes in patients with nonsevere COVID-19. Our study observed that approximately one-fifth of the participants exhibited liver enzyme abnormalities at their 6-month follow-up visit. Among these, 12.2% had persistently high levels of AST, ALT, or both. Consistent with our observations, a study in Shenzhen, China, reported that 10% of patients with severe or nonsevere COVID-19 had abnormal AST to ALT ratios 40 days after discharge^56^. These results highlight the importance of monitoring long-term hepatic abnormalities in patients with nonsevere COVID-19, particularly those at high risk. However, the underlying causes of liver abnormalities are likely multifactorial and warrant further investigation.

Recent studies have shown that SARS-CoV-2 strongly stimulates human immunity, hyperinflammation, and cytokines^10,57^. CRP, one of the acute phase proteins produced by liver cells, is associated with the severity of infection, acute inflammation, and chronic inflammation^58^. In patients with COVID-19, CRP levels could be used to predict severe pneumonia^59^. CRP levels significantly surged in severely SARS-CoV-2-infected patients, but levels fell slightly once the virus was eliminated^60^. A previous investigation found that 9.5% to 16.0% of individuals who recovered from COVID-19 still had high CRP levels (≥ 5 mg/L) in the second month after hospital discharge^61,62^. Similarly, our study demonstrated that in healthy and high-risk hosts, 14.8% of nonsevere cases had persistently high CRP levels (≥ 5 mg/L) 6 months after COVID-19. This observation accords with earlier studies^63,64^ that found that patients with COVID-19 who were metabolically ill with obesity and diabetes showed significantly elevated CRP levels. We hypothesize that the long-term multiple metabolic abnormalities in our cohort population might explain the persistence of the elevated CRP levels in both host groups. In the case of the high-risk hosts, the mean CRP level was double that of the healthy hosts at 3 months. Despite a subsequent decrease in both host groups’ levels, the high-risk hosts’ mean CRP level was still greater than that of the healthy hosts at 6 months. This finding also supports previous evidence that SARS-CoV-2 stimulates the inflammatory process not only during the acute phase of infection but also in the period 3–6 months after infection. The relationship between metabolic abnormalities and CRP levels should be investigated further.

Our analysis focused on long-term multiple metabolic abnormalities after nonsevere SARS-CoV-2 infection. Being a healthy host, being female, having dyslipidemia, and being fully vaccinated are protective factors against worsening long-term multiple metabolic abnormalities. Interestingly, dyslipidemia is a protective factor against metabolic complications. This finding might be because the people diagnosed with dyslipidemia before their COVID-19 infection had already received lipid-lowering medications and critical information that had promoted healthy lifestyle changes. The relative protective effects of women and men against the long-term metabolic consequences after nonsevere COVID-19 were evident in our study. Consistent with our observations, other studies reported a relatively higher number of deaths from COVID-19 in men than in women. Those studies investigated the outcomes in the general population and diabetic patients^65,66^. It has been previously hypothesized that there are potential gender-specific mechanisms modulating the natural course of COVID-19 consequences. These mechanisms include the hormone-regulated expression of genes encoding ACE2; sex hormone-driven immune responses; sex-specific aspects of antiviral therapies; and the impacts of sex-specific lifestyles, health behaviors, and socioeconomic conditions on COVID-19^65^. However, the definitive mechanisms behind sex and the risk of multiple metabolic abnormalities remain to be investigated.

Our study should be interpreted in light of several strengths and some limitations. This is the first prospective study to investigate several components of long-term metabolic outcomes. The follow-up period was up to 6 months. Furthermore, we explored which variations in clinical parameters are related to long-term metabolic abnormalities in Thai patients with nonsevere COVID-19. Second, the number of participants in each of our cohorts is acceptable, and the follow-up duration is longer than those used in previous studies of nonsevere cases of COVID-19.

The main limitation of our study was the need for more clinical data: body weight before the onset of COVID-19 and some laboratory information before and upon the onset of COVID-19. This absence is attributed to the standard-care procedures for nonhospitalized patients with COVID-19. However, the investigators made efforts to obtain all available information from the hospital’s database records and through interviews with the participants during follow-up visits. Second, the data collected were derived from nonfasting blood samples or measurements taken in the nonfasting state. Consequently, the present study did not evaluate some parameters: body composition in the fasting state, fasting plasma glucose, triglycerides, and high-density lipoprotein cholesterol. Third, although corticosteroids may impact body weight and glucose levels, only a small proportion of our cohort received out-of-hospital, short-term dexamethasone treatment. This therapy likely had a negligible effect on their long-term weight and metabolic abnormalities. Lastly, the metabolic abnormalities among patients with non-severe COVID-19 are probably complex and multifactorial. Therefore, more detailed information on individual characteristics would have been of value, particularly data on diet, physical activity, alcohol use, smoking, mental and emotional health, anti-inflammatory substances, and current medications. Such characteristics may have interfered with our metabolic and CRP results. Moreover, the magnitude and the difference of worse metabolic outcomes between participants with and without COVID-19 cannot be adequately evaluated without matched contemporary controls.

Our key finding was that more than one-third of the healthy individuals and nearly half of the high-risk participants with nonsevere COVID-19 had multiple long-term metabolic abnormalities, particularly in glycemia and lipids. We also demonstrated that being a male, being a high-risk host, and receiving fewer than 3 doses of any COVID-19 vaccine are independently associated with multiple long-term metabolic consequences. All individuals with nonsevere COVID-19, even healthy hosts, should be advised to adopt healthy lifestyles and have appropriate clinical follow-ups. Further work is needed to confirm and explain the mechanisms behind metabolic abnormalities in post-COVID-19 patients.

## Materials and methods

### Study design and participants

**Figure 6:**
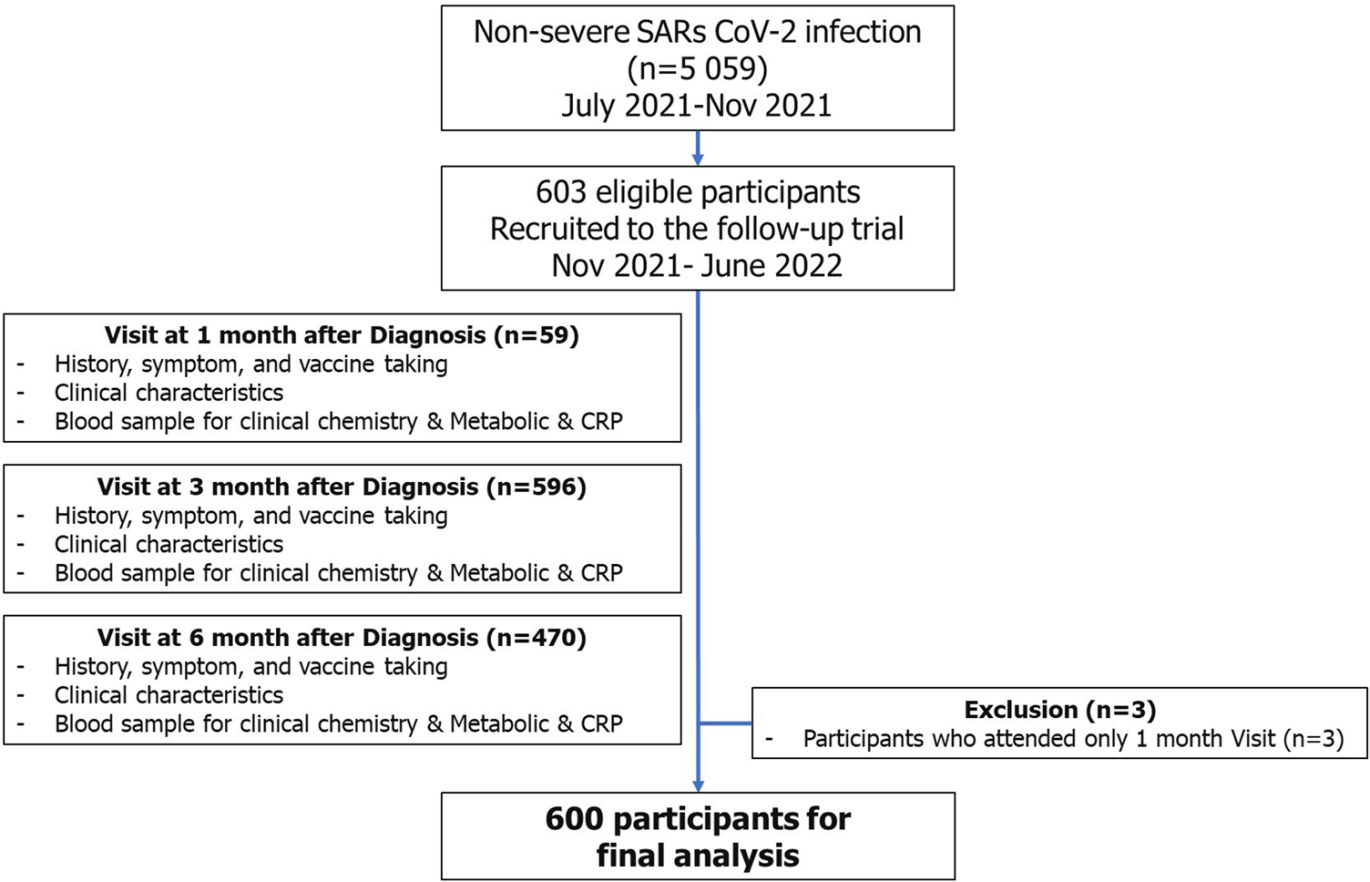
Enrollment of the study population.

Since July 2021, HI and Hospitals have been available in Thailand as alternative means of caring for people with nonsevere COVID-19. In all, 2711 and 2348 individuals with nonsevere COVID-19 were registered in the HI and hospital systems, respectively. While both isolation strategies were functioning, the Delta variant (B.1.617.2) wave of coronavirus swept through Thailand. Consequently, our prospective observational cohort study recruited participants affected by the Delta lineage. We invited individuals to participate if they had nonsevere COVID-19 and were registered in HI or a hospital between August and November 2021. Their enrollment and follow-up visits occurred at Siriraj Hospital, Bangkok, 1, 3, and 6 months after symptom onset (i.e., between November 2021 and June 2022). Details of the patients’ clinical characteristics, blood collections, and any procedures conducted during the visits are illustrated in Fig. 6. Before this research began, its protocol was approved by the Siriraj Institutional Review Board (COA SI 732/2021), Faculty of Medicine Siriraj Hospital, Mahidol University, Bangkok, Thailand. All methods were carried out in accordance with relevant guidelines and regulations.

The study enrolled people aged at least 18 years with nonsevere COVID-19. Patients were screened by preliminary clinical evaluation, and COVID-19 infections were confirmed by real-time reverse transcription polymerase chain reaction. All participants were asymptomatic or mildly symptomatic, and none needed oxygen supplementation, intravenous medications, or hospitalization at the onset of presentation. Patients were excluded if they were pregnant or unwilling to participate in follow-up visits. Participants were only included in the study’s analyses if they had attended at least the 3- or 6-month follow-up visit.

### Procedures and measurements

Information on the eligible patients was obtained from relevant medical records. Data before and during infection were collected. They comprised the patients’ demographic profiles, clinical characteristics, immunization statuses, comorbidities, clinical outcomes, anthropometric measurements (height and weight), and laboratory findings during HI or while in a hospital. These data were confirmed by the participants, face to face, at their first follow-up visit. At each follow-up session, the patients were interviewed to obtain data on their remaining or new symptoms, any newly diagnosed comorbidities, changes to their immunization status, and current body weight. Each participant’s health-related quality of life was also assessed by trained staff using the EQ-5D-5L questionnaire. Utility scores were then calculated using coefficient values specific to the Thai population^67^.

Body mass index (BMI) was calculated by weight (kg) divided by squared height (m^2^) and categorized into 3 levels: BMI < 25, 25–< 30, and ≥ 30 kg/m^2^. Blood samples were taken in the nonfasted state to determine glycated hemoglobin (HbA1c), direct low-density lipoprotein cholesterol (dLDL-c), aspartate transaminase (AST), alanine aminotransferase (ALT), and C-reactive protein (CRP) levels. HbA1c was measured by a high-performance liquid chromatography system using a Tosoh G8 HPLC Analyzer (Tosoh Bioscience Inc, San Francisco, CA, USA). The approved methodology and standardizations of the International Federation of Clinical Chemistry were followed for the blood sample analyses. A particle-enhanced immunoturbidimetric assay measured CRP. Standard laboratory procedures determined AST, ALT, and dLDL-c levels.

### Definitions and outcome measures

Participants were divided into 2 groups based on their risks for COVID-19 complications: “high-risk hosts” and “healthy hosts”. High-risk hosts were those with at least 1 of the following risks: age ≥ 60 years, BMI ≥ 30 kg/m^2^, chronic lung disease (chronic obstructive pulmonary disease or asthma), chronic kidney disease, coronary heart disease, cerebrovascular disease, diabetes, and active malignancy. Healthy hosts were those without these risks or comorbidities^68^.

Participants were deemed to have “long-term multiple metabolic abnormalities” at the 6-month follow-up if they still had a metabolic abnormality or their abnormalities had worsened to “significant.” Significant abnormalities were indicated by the presence of any 2 or more of the following:

- Abnormal BMI. This was (1) the worsening of BMI to at least 1 higher BMI category or (2) maintaining a high BMI (≥ 30 kg/m^2^) but with an increase in the BMI value.
- Abnormal glucose homeostasis. This was (1) an HbA1c of 5.7–6.4%, (2) newly diagnosed diabetes, or (3) known diabetes with an HbA1c ≥ 9%.
- Abnormal lipids. This was (1) previously diagnosed dyslipidemia with persistent dLDL-c ≥ 130 mg/dL or (2) newly detected hypercholesterolemia (dLDL-c ≥ 130 mg/dL) that persisted to the 6-month follow-up.
- Abnormal liver function. This was (1) the worsening of AST, ALT, or both to > 30 U/L, or (2) persistently abnormal levels of AST, ALT, or both (≥ 30 U/L).
- Abnormal CRP. This was (1) a persistently abnormal CRP level (≥ 5 mg/L) or (2) the CRP level worsening to ≥ 5 mg/L.

The participants’ baseline diabetes statuses before the onset of COVID-19 were determined from their medical records or were self-reported during their follow-up interviews. Cases of newly diagnosed diabetes were ascertained from medical documents that recorded the diagnosis of diabetes by a doctor, an oral hypoglycemic agent, and an HbA1c level ≥ 6.5% after the onset of COVID-19. The diabetes statuses of the participants were stratified into the following 4 groups according to American Diabetes Association guidelines^69^:

- “nondiabetic”: HbA1c < 5.7%
- “prediabetes”: HbA1c > 5.7% but ≤ 6.4%
- “newly diagnosed diabetes”: HbA1c ≥ 6.5% or use of diabetes medications after the onset of COVID-19 infection
- “known diabetes”: known diagnosis of diabetes or current use of diabetes medications before the onset of the COVID-19 infection

Regarding the lipid metabolic component, newly diagnosed hypercholesterolemia was defined by having no documented history of dyslipidemia prior to COVID infection, with a dLDL level ≥ 130 mg/dL. Patients with a dLDL level ≥ 190 mg/dL were deemed to have severe hypercholesterolemia. Uncontrolled LDL cholesterol (LDL-c) was defined as a known history of dyslipidemia or a current statin prescription with dLDL-c ≥ 130 mg/dL.

Immunization status before the onset of COVID-19 was categorized as follows:

- “not immunized”: people without COVID-19 vaccinations and those vaccinated within 14 days before COVID-19 onset
- “partially immunized”: people with only 1 vaccine dose more than 14 days before COVID-19 onset
- “fully immunized”: people with at least 2 vaccine doses more than 14 days before COVID-19 onset

The primary outcomes of this study were the prevalence of long-term multiple metabolic abnormalities 6 months after the onset of infection in people with nonsevere COVID-19. The secondary outcomes were the factors associated with the multiple metabolic abnormalities and the prevalence of each component of the long-term metabolic abnormalities in people with nonsevere COVID-19.

### Statistical analysis

Since there is no previous report on the prevalence of long-term multiple metabolic abnormalities in people with nonsevere COVID-19, we could not determine the sample size for our investigation based on existing evidence. However, we did calculate the statistical power to assess the primary outcome using the results of our samples. If we accepted a prevalence of long-term multiple metabolic abnormalities within ± 10% of the true value in the population (prevalence = 31.3–51.3%) and set α to 0.05, the power of our study would be 99%. Additionally, at α = 0.05, the power to detect the difference in prevalence in healthy hosts (36.5%) vs high-risk hosts (49.2%) would be 77%.

Statistical analyses were performed with IBM SPSS Statistics for Windows, version 26.0 (IBM Corp, Armonk, NY, USA). Normally distributed continuous variables are given as the means ± standard deviations, while continuous variables with skewed distributions are shown as medians with interquartile ranges. Categorical variables are expressed as absolute values and percentages. The data of the high-risk and healthy hosts are reported separately. The clinical characteristics, blood chemistry data, and long-term metabolic abnormalities of the 2 groups are presented as the mean changes and changes in the categories of metabolic abnormalities during the follow-up period.

For the statistical comparisons of clinical characteristics, the blood chemistry data and long-term metabolic abnormalities of the 2 distinct groups (i.e., healthy hosts versus high-risk hosts, and people with no or mild metabolic abnormalities versus those with multiple metabolic abnormalities), the unpaired t-test, Mann–Whitney U test, χ^2^ test, Fisher’s exact test, and McNemar–Bowker test were used, as appropriate. The odds ratio (OR) was calculated using a logistic regression model in which OR was equal to the exponentiated regression coefficient. For within-subject comparisons of the metabolic abnormality categories at 3 months vs 6 months, we used the Wilcoxon signed-rank test and McNemar–Bowker test, as appropriate. Comparisons of the mean of each metabolic change between follow-up visits were performed using repeated-measures ANOVA with Bonferroni adjustment for multiple comparisons. Finally, the clinical characteristics, comorbidities, COVID-19 symptoms, immunization statuses, utilities, and scores from the quality of life with a visual analog scale^70^ of participants with and without multiple metabolic abnormalities were compared. We used multivariable logistic regression analysis to explore the associations between the potential factors and multiple metabolic abnormalities.

### Informed consent

Informed consent was obtained from all subjects involved in the study.

### Supplementary Information

Supplementary Information.

## Data Availability

The data presented in this study are available on request from the corresponding author. The data are not publicly available due to privacy and ethical reasons.
