# Supplementary material for: Long-term multiple metabolic abnormalities among healthy and high-risk people following nonsevere COVID-19
Source: Sci Rep. 2023 Aug 31;13:14336. doi: 10.1038/s41598-023-41523-5 (PMC10471587; doi:10.1038/s41598-023-41523-5)
Supplement: Supplementary file 1 — Supplementary Information. [file 41598_2023_41523_MOESM1_ESM.docx]

# Supplementary Material for the manuscript entitled *“Long-term multiple metabolic abnormalities among healthy and high-risk people following non-severe COVID-19”*

**Supplementary Table 1.** Clinical characteristics, comorbidities, and immunization statuses of people with non-severe COVID-19 with and without multiple metabolic abnormalities

| **Baseline parameter** | **No or mild**  **metabolic abnormalities**  **(n=273)** | **Multiple metabolic abnormalities**  **(n=192)** | ***P*** |
| --- | --- | --- | --- |
| Female, n (%) | 183 (67.0%) | 105 (54.7%) | 0.007 |
| Age, mean (±SD) | 45.7±14.9 | 46.5±13.9 | 0.5 |
| BMI, mean (±SD) | 24.2±4.7 | 27.5±5.0 | <0.001 |
| N-gene CT threshold | 21.7±5.5 | 21.1±5.6 | 0.2 |
| Utility score (median, IQR) | 0.89 (0.71, 0.94) | 0.88 (0.73, 0.94) | 0.9 |
| VAS score (median, IQR) | 70 (50, 90) | 75 (50, 85) | 0.5 |
| Symptomatic at presentation, n (%) | 241 (88.3%) | 176 (91.7%) | 0.2 |
| HT, n (%) | 76 (27.8%) | 46 (24.0%) | 0.3 |
| DLP, n (%) | 58 (21.2%) | 21 (10.9%) | 0.004 |
| Recovery at home, n (%) | 257 (94.1%) | 181 (94.3%) | 0.9 |
| Immunization status, n (%) |  |  | 0.05 |
| - Not immunized | 87 (31.9%) | 75 (39.1%) |  |
| - Partially immunized | 98 (35.9%) | 72 (37.5%) |  |
| - Fully immunized (2 doses) | 62 (22.7%) | 38 (19.8%) |  |
| - Fully immunized (3 doses) | 26 (9.5%) | 7 (3.6%) |  |
| Vaccine booster within 6 m after COVID-19, n (%) |  |  |  |
| - No vaccine booster | 22 (8.1%) | 26 (13.5%) | 0.07 |
| - Partial vaccine booster <14 days | 6 (2.2%) | 8 (4.2%) |  |
| - 1 booster dose | 166 (60.8%) | 98 (51.0%) |  |
| - 2 booster doses | 79 (28.9%) | 60 (31.3%) |  |
| Overall host status |  |  |  |
| - Healthy hosts, n (%) | 183 (67.0%) | 105 (54.7%) | 0.007 |
| - High-risk hosts | 90 (33.0%) | 87 (45.3%) |  |

DLP, dyslipidemia; HT, hypertension; IQR, interquartile range; m, month; SD, standard deviation
